# Development of the Vestibular Lamina in Human Embryos: Morphogenesis and Vestibule Formation

**DOI:** 10.3389/fphys.2020.00753

**Published:** 2020-07-16

**Authors:** Tengyang Qiu, Tathyane H. N. Teshima, Maria Hovorakova, Abigail S. Tucker

**Affiliations:** ^1^Centre for Craniofacial and Regenerative Biology, Faculty of Dentistry, Oral and Craniofacial Sciences, King’s College London, London, United Kingdom; ^2^Institute of Histology and Embryology, First Faculty of Medicine, Charles University in Prague, Prague, Czechia; ^3^Institute of Experimental Medicine, Czech Academy of Sciences, Prague, Czechia

**Keywords:** dental pathologies, keratin, epithelial differentiation, apoptosis, oral mucosa, human development

## Abstract

The vestibular lamina (VL) is a transient developmental structure that forms the lip furrow, creating a gap between the lips/cheeks and teeth (oral vestibule). Surprisingly, little is known about the development of the VL and its relationship to the adjacent dental lamina (DL), which forms the teeth. In some congenital disorders, such as Ellis-van Creveld (EVC) syndrome, development of the VL is disrupted and multiple supernumerary frenula form, physically linking the lips and teeth. Here, we assess the normal development of the VL in human embryos from 6.5 (CS19) to 13 weeks of development, showing the close relationship between the VL and DL, from initiation to differentiation. In the anterior lower region, the two structures arise from the same epithelial thickening. The VL then undergoes complex morphogenetic changes during development, forming a branched structure that separates to create the vestibule. Changing expression of keratins highlight the differentiation patterns in the VL, with fissure formation linked to the onset of filaggrin. Apoptosis is involved in removal of the central portion of the VL to create a broad furrow between the future cheek and gum. This research forms an essential base to further explore developmental defects in this part of the oral cavity.

## Introduction

The vestibular lamina (VL) or lip furrow band is an embryonic structure that forms the oral vestibule (vestibulum oris), the gap in between the teeth and cheeks and lips. The oral vestibule has been proposed to have evolved to aid suckling, and as such is assumed to be a structure unique to mammals and the evolution of lactation. The VL develops labially/buccally to the dental lamina (DL), which forms the dentition of the jaw. These two laminas have been suggested to originate from a common oral epithelial thickening, which subdivides into the two laminas in the mouse (Peterková, 1985). The thickening of the oral epithelium occurs approximately during the 6th week in human embryos, with a single lamina suggested to split to form two diverticula (Bolk, 1921). However, two independently forming laminas have also been described, with the VL forming before or after the DL (Schour, 1929; Tonge, 1969; Nery et al., 1970). The various theories are summarized in Hovorakova et al. (2005). Tooth buds along the DL progress through bud, cap, and bell stages (Tucker and Sharpe, 2004), while the neighboring VL opens up to create a cleft separating the lips and checks from the dental arch between the 12th and 14th week (Coslet and Cohen, 1969). Although the VL and DL have a very close early relationship, the VL is largely ignored in odontogenetic studies.

**Figure 1:**
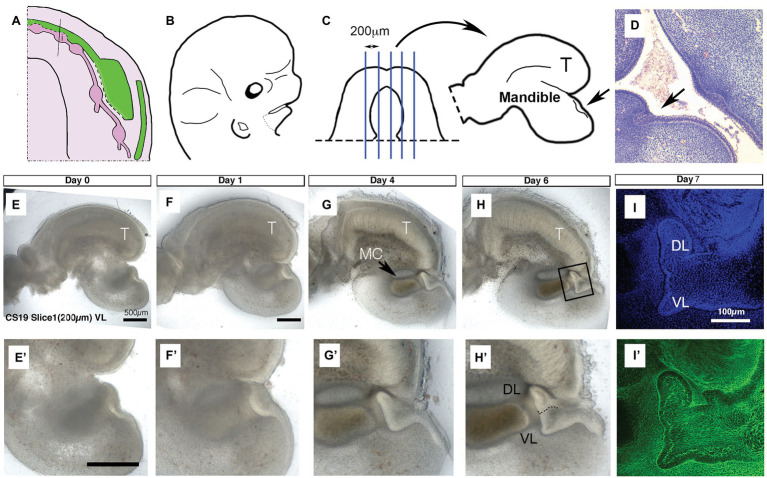
United development of the vestibular lamina (VL) and dental lamina (DL) in the anterior mandible. **(A)** Schematic of the developing VL (green) and DL (pink) during human embryonic lower jaw development. Line through deciduous incisor (i) tooth germ indicates the plane of dissection for culture. **(B)** Schematic of head at CS19 (46 days). Dots indicate dissection of lower jaw. **(C)** Schematic of dissected lower jaw, with blue lines indicating chopping planes to create sagittal live slices. Medial slices contain tongue (T) and single thickening for the DL/VL (arrow). **(D)** Histology section through an embryonic head at 6 weeks. Arrow points to single thickening for the DL/VL. **(E–H)** Developing live sagittal slices of a CS19 mandible. **(E’–H’)** Close up of developing VL/DL area shown in **(E–H)**. **(E,E’)** Day 0. A single epithelial thickening is evident under the tongue (T). **(F,F’)** Day 1. **(G,G’)** Day 4. Meckel’s cartilage (MC) has condensed and differentiated during the culture period. The tongue (T) has started to fuse with the mandible underneath as an artifact of the culture system. **(H,H’)** Day 6. **(I,I’)** Day 7 slice fixed and processed for immunofluorescence. Boxed inset in **(H)** shows equivalent regions highlighted in **(I,I’)**. **(I)** DAPI, **(I’)** Phalloidin stain highlighting F-actin. Schematic in **(A)** based on data in the literature (Hovorakova et al., 2005, 2007). Scale bar in **(E,E’,F)** = 500 μm, same scale in **(G,H)** and **(F’–H’)**. Scale bar in **(I)** = 100 μm, same scale in **(I’)**. VL, vestibular lamina; DL, dental lamina; T, tongue; MC, Meckel’s cartilage.

**Figure 2:**
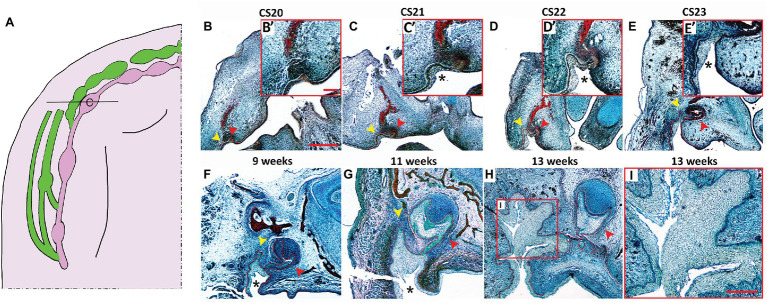
Histological development of the VL and DL in the upper canine region. **(A)** Schematic of the developing VL (green) and DL (pink) during human embryonic upper jaw development. Line through deciduous canine (c) tooth germ indicates the position of the sections in **(B–I)**. **(B–I)** Frontal sections human embryo and fetal tissue stained with a trichrome stain (Alcian blue, Alizarin red, and haematoxylin). **(B)** CS20, **(C)** CS21, **(D)** CS22, **(E)** CS23, **(F)** 9 weeks, **(G)** 11 weeks, **(H,I)** 13 weeks. High power view of VL/DL shown in red inset **(B’–E’)**. Boxed area in (H) shown in (I). DL and VL outlined by red dashed lines in **(B–H)**. Schematic in **(A)** based on data in the literature (Hovorakova et al., 2005, 2007). Yellow arrowheads indicate forming VL. Red arrowheads indicate forming DL. ^*^ = cheek furrow or labio-tectal furrow, which forms from CS21 at the canine. Scale bar in **(B)** = 500 μm, same scale in **(C–H)**. Scale bar in inset **(B’)** = 125 μm, same scale in insets **(C’,D’,E’)**. Scale bar in **(I)** = 250 μm.

Originally the relationship between the human embryonic VL and DL was represented in a classical “horseshoe-shape,” with the VL running in parallel on the outside of the DL. This view was based on histological sections of the upper jaw, where the VL appeared continuous throughout the jaw. Interestingly, however, when viewed in 3D, the human VL is clearly discontinuous, integrating with the DL at distinct points along the jaw, and is therefore a much more complex structure than initially proposed (Figures 1A, 2A; Hovorakova et al., 2005, 2007). The complex relationship of the VL and DL in different parts of the jaw probably explains the different accounts of their development, and it appears likely that, at least in the anterior region of the human lower jaw, the VL and DL do share a common origin (Bolk, 1921; Hovorakova et al., 2007). This common origin of the VL and DL is supported by the discovery that both laminas in the anterior area form from a common Shh expressing domain in mice (Hovorakova et al., 2016).

The VL has been described in sheep, mouse, humans, and voles (Pavlikova et al., 1999; Hovorakova et al., 2005, 2016; Witter et al., 2005). Across species, clear anatomical differences are evident along the jaw. For example, in the mouse the VL is very prominent in the anterior lower jaw, while it is almost absent in the anterior upper jaw. The mouse and vole have very thin VLs, while the sheep and human have very thick multi-layered VLs. Such differences are likely to lead to differences in morphology of the final oral cavity and may reflect differences in diet.

The early VL has similarities to other embryonic structures that have a close relationship to the teeth across vertebrates. For example, in reptiles the DL shares a common origin with the adjacent forming dental glands (Vonk et al., 2008; Tucker, 2010). Here, a single epithelial thickening appears to divide to form the DL and a gland, similar to the process described for the VL and DL (Kochva, 1965). Both the VL of mammals and the dental gland primordium of reptiles form on the outside of the DL and potentially evolved from an outer tooth row from a common amniote ancestor (Hovorakova et al., 2020). This theory is supported by the existence of tooth-germ like bulges that transiently form in the upper VL (Figure 2A) and by the high incidence of odontomas and other dental pathologies in the oral vestibule (Hovorakova et al., 2020). In animal models, the VL has the potential to form tooth-like odontomas when signaling in this tissue is perturbed. In mice, supernumerary teeth have been reported to form in the VL region after overexpression of the Wnt pathway in adenomatous polyposis coli conditional knockouts (Wang et al., 2009) and after stabilization of β-catenin in Sox2^+^ positive cells (Popa et al., 2019). The close developmental and evolutionary relationship between the VL and DL may, therefore, explain some of these pathologies.

In addition, the VL is one of the most affected oral tissues in Ellis-van Creveld (EVC) syndrome patients, where defects disrupt the VL development and lead to fusion of the upper lip to the gingiva with multiple frenula (Sasalawad et al., 2013). However, little is known about the development of the VL. We therefore investigated the development of the VL and DL in human embryos and assessed the mechanisms of fissure formation in the vestibular epithelium as the oral vestibule forms. We used organ culture, histology, and immunofluorescence to study the VL development at different stages of human embryos. We have observed that the VL and DL have a very close relationship. We show the complex structure of the VL during development and highlight the roles of epithelial differentiation, proliferation, and apoptosis in the opening of the VL in human embryos. These findings can help our understanding of the normal development of the VL and shed light on the complex relationships between the VL and DL during development, which is crucial for the further exploration of developmental VL defects.

## Materials and Methods

Human embryos/fetuses were provided by the Human Developmental Biology Resource (HDBR; Project 200504: Characterizing the development of the oral vestibular lamina). Eight stages of human development were examined to study the development of the VL. Samples included CS19 (46 days), CS20 (49 days), CS21 (51 days), CS22 (53 days), CS23 (56 days), 9, 11, and 13 weeks stages. *N* = 1–2 freshly fixed samples for immunofluorescence were analyzed at each stage (total *N* = 8). HDBR samples were compared with a larger archival histology collection from the Department of Teratology, IEM, CAS, Prague compiled from the 1960’s to the 1980’s (recently deposited at First Medical Faculty, Charles University, Prague; *N* = 53 in total ranging from CS17 to 9 weeks). The stages in the study were selected as they span the period from the initiation of the VL and DL to fissure formation in the VL (Coslet and Cohen, 1969; Hovorakova et al., 2005).

### Slice Culture

The lower jaw was isolated from one CS19 and one CS20 human embryo and chopped sagittally at a cutting distance of 200 μm using a McIlwain tissue chopper (Alfaqeeh and Tucker, 2013). A clear DL/VL bud was only evident in the CS19 specimen, so this was used for further analysis for this project. Selected slices from the lower jaw were placed on permeable membranes (BD) over culture medium [DMEM-Advanced Dulbecco Modified Eagle Medium F12, (Invitrogen); 1% GlutaMAX (Invitrogen); and 1% penicillin-streptomycin solution (10,000 units penicillin and 10 mg streptomycin/ml; Sigma-Aldrich)]. Slices (*N* = 3 from CS19) were photographed by using a Leica dissecting microscope at day 0 of culture, to record the morphology, and then incubated in 5% CO_2_ at 37°C with the culture medium changed every 2–3 days. Slices were photographed at regular intervals for 7 days before fixation in 4% paraformaldehyde (PFA).

### Tissue Processing and Histology

The upper jaws of heads were dissected from human embryos (CS20, CS21, CS22, CS23, 9, 11, and 13 weeks) and fixed in 4% PFA. Calcified tissues were decalcified in 0.5 M ethylenediaminetetraacetic acid (EDTA) in PBS. After decalcification, samples were dehydrated in an increasing ethanol concentration and permeated in xylene. Samples were then embedded in paraffin and cut in 10 μm serial sections by Microtome Leica RM2245. One of the sections in the series was stained with trichrome staining (Sirrus red, Alcian blue, and hematoxylin). Stained slides were observed under the Nikon Eclipse 80i light microscope, and images were taken by the attached Nikon Digital Sight DS-Fi1 camera.

### Immunofluorescence

Wax embedded serial sections of the VL were de-waxed, rehydrated, and transferred into the citric acid (pH = 6) in 92°C water bath for antigen retrieval. The antibody blocking solution consists of PBS, 0.05% Tween20, 10% goat serum, and 1% bovine serum albumin (1% = 1 g/100 μl). The slides were then incubated with rabbit keratin 14 (K14; 1:200; Covance #905501), mouse keratin 10 (K10; 1:300; Abcam #ab76318), rabbit keratin 5 (K5; 1:300; Covance #PRB-160P), rabbit fillagrin (Cambridge Bioscience #HPA030188), rabbit proliferating cell nuclear antigen (PCNA; Abcam #ab193965), and rabbit Cleaved Caspase-3 (1:200, Cell Signaling #9579) overnight at 4°C. For immunofluorescence, sections were incubated in Alexa Fluor™ donkey anti-mouse 488 (1:500; Invitrogen #A11001), Alexa Fluor™ donkey anti-rabbit 488 (1:500; Abcam ab150073), and Alexa Fluor™ donkey anti-rabbit 568 (1:500; Invitrogen #A10042) for 2 h at RT. Sections were mounted with Fluoroshield™ with DAPI (Sigma-Aldrich #SLBV4269) and imaged with a Leica TCS SP5 confocal microscope or Zeiss ApoTome. To test each antibody, controls were performed where the primary antibodies had been omitted in order to confirm specific staining. Each antibody was repeated at least twice, at different timepoints, using serial sections. To aid comparison, the color of filaggrin was changed to green from red on the ApoTome (13 weeks) or in photoshop (11 weeks), while the K5 was changed on the ApoTome from red to blue.

For wholemount immunofluorescence, explant culture slices were fixed in 4% PFA for 30 min at RT. Samples were permeabilized with PBS Triton 0.5% (PBT) at RT for 1 h, followed by trypsinization for 5 min on ice and incubation in blocking solution for 2 h. After blocking, slices were incubated in Alexa Fluor Phalloidin 488 (Invitrogen; 1:50) and DAPI (1:1,000; Sigma) overnight at 4°C. After washing in PBS, cultures were mounted in glycerol and analyzed by a Leica TCS SP5 confocal microscope.

## Results

### The Human VL and DL Bud Off From a Single Epithelial Thickening

It has been suggested from 3D reconstructions of histology sections that the human VL and DL are derived from a common oral epithelium in the lower lip region (Figure 1A; Hovorakova et al., 2007). To follow the development of these two laminas during development, we made live slices through the dissected mandible of a human embryo at the placode stage (CS19: 6.5 weeks; Figures 1B,C; *N* = 1 embryo). Slices were sectioned in the sagittal plane, and medial slices that contained an epithelial thickening under the developing tongue were selected (*N* = 3 slices). A single thickening was observed at CS19 (Figures 1E,E’), similar to that observed in histology sections through this region of the jaw (Figure 1D). The thickening in culture became more pronounced after a day in culture (Figures 1F,F’). The thickening extended into the underlying mesenchyme, with a lip of epithelial cells elongating toward the tongue after 4 days (Figures 1G,G’). The epithelium divided into two protrusions after 6 days in culture forming the VL and DL (Figures 1H,H’). At day 7, a clear bifurcation of the epithelium into a tooth bud and a wider VL was observed when imaged by confocal, with phalloidin staining (green) used to highlight the cell morphology (Figures 1I,I’). This confirms the histology findings that, at least in the anterior region of the lower jaw, the human VL and DL form from a single placode and are therefore directly associated with each other.

### The Morphology of the Human VL Increases in Complexity During Development

To further investigate the development of the VL and DL in human embryos, we studied the development of VL and DL at embryonic CS20, CS21, CS22, CS23, 9, 11, and 13 weeks. This time period spans the period from defined VL and DL thickenings to proposed formation of the fissure in the VL to create the oral vestibule (Coslet and Cohen, 1969). The relationship between the VL and DL is very complex across the jaw with large variations depending on the A-P position (summarized in Figures 1A, 2A). In order to follow how the VL and DL co-develop, we therefore focused on a single region in the jaw. For this, we selected the deciduous canine area of the maxilla. The upper canine and VL are very closely associated in 3D reconstructions (Figure 2A), and the VL in this region appears particularly complex in shape (Bolk, 1921, data not shown), thus providing an intriguing area to study further. The primary canine primordia at these stages develop from an oral epithelial thickening at CS20 (Figures 2B,B’), bud stage at CS21 (Figures 2C,C’) and CS22 (Figures 2D,D’), to cap stage at CS23 (Figures 2E,E’) and 9 weeks (Figure 2F), to a bell stage at 11 (Figure 2G) and 13 weeks (Figure 2H). At CS20, the VL was visible as a slight thickening on the buccal side of the tooth germ, forming a prominent thickening by CS21 (Figures 2B–C’). By CS22, the whole VL/DL region had invaginated inward to sit within a groove (Figures 2D,D’). This groove has previously been described as the labio-tectal furrow and the cheek furrow (Bolk, 1921; Hovorakova et al., 2005). The cheek furrow deepened as the VL/DL developed, with the cap stage canine tooth connected to the VL (Figures 2E,F). A narrow fissure in the VL started to form at 11 weeks, extending up from the base of the furrow (Figure 2G). By 13 weeks, several fissures had formed that split the VL into a branched structure, with the DL extending from the lingual side of the VL (Figures 2H,I).

### Epithelial Differentiation Highlights Labial-Lingual Differences in Structure of the VL and May Trigger Furrow Formation

**Figure 3:**
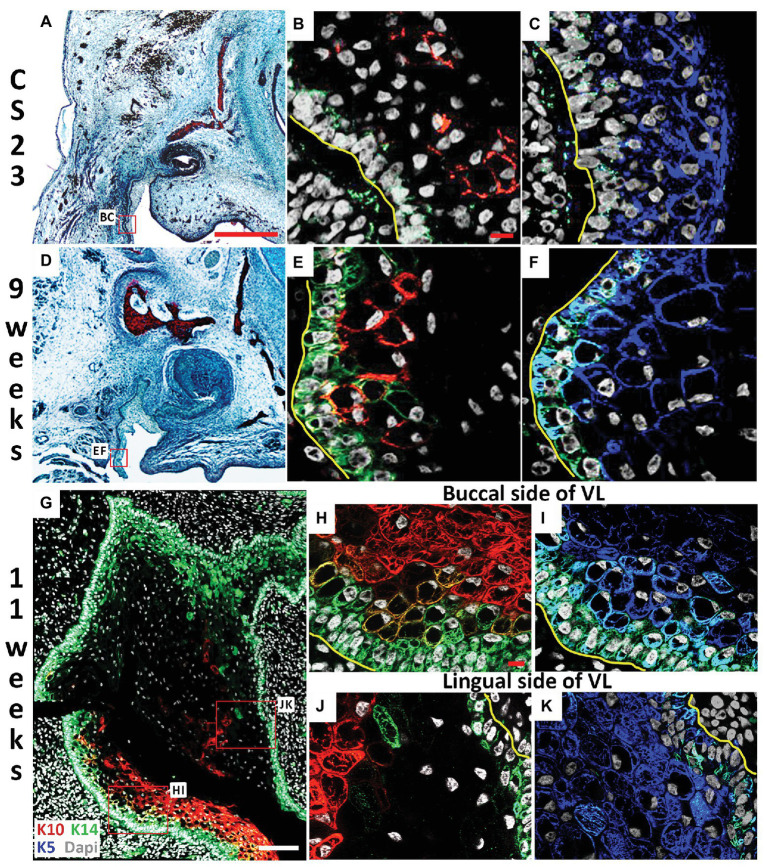
Lingual/buccal differences in VL epithelium. **(A,D)** Histological frontal sections through the upper canine. **(B,C,E–K)** Immunofluorescence. DAPI shows nuclei in white. **(A–C)** CS23. Box in **(A)** highlights regions in **(B,C)**. The buccal side of the cheek furrow comprises cells with **(B)** limited Keratin 14 (K14; Green) and Keratin 10 (K10; red), and **(C)** high levels of Keratin 5 (K5; blue). **(D–F)** 9 weeks. Box in **(D)** highlights regions in **(E,F)**. The buccal side of the cheek furrow comprises cells with **(E)** robust K14 in the basal layer (Green), with K10 turning on in the overlying suprabasal layer (red), and **(F)** high levels of K5 (blue). **(G–K)** 11 weeks. Box in **(G)** highlights regions in **(J,K)**. **(G)** K14 (green) is robustly expressed around the edges of the VL, with K10 (red) observed at high levels mainly on the buccal side only. **(H–K)** High power of the buccal side **(H,I)** and lingual **(J,K)** side of the VL comparing the expression of K5 (blue) and K14 (green) in the basal layer and overlying layer, and K10 (red) in the suprabasal layer. Yellow lines outline the basement membrane separating the epithelial and mesenchymal cells in **(B,C,E,F,H–K)**. Scale bars in **(A)** = 500 μm, same scale in **(D)**. Scales bars in **(B,H)** = 10 μm, same scale in **(C,E,F,I–K)**. Scale bar in **(G)** = 100 μm.

Skin epidermal differentiation occurs in layers from the basal cell layer, where the basal cells sit on the basement membrane adjacent to the surrounding mesenchymal cells, to the superficial cornified layer. K5 and K14 are expressed in more undifferentiated/basal cells; while K10 is expressed in the middle spinous layer with filaggrin expressed in the overlying granular layer (Lee et al., 1999; Coolen et al., 2010). We therefore utilized K5, K14, K10, and filaggrin as markers to investigate the differentiation state of the VL from CS23 to 13 weeks. The expression of various keratins have previously been followed in the human oral mucosa, with expression of K10 in the VL, but not DL, shown at 11 weeks (Pelissier et al., 1992). Where, it was suggested that the boundary between the K10 positive and negative cells in the VL might indicate the site of future fissure formation (Pelissier et al., 1992). At CS23, the buccal oral epithelium within the labio-tectal furrow expressed K5, with very limited expression of K10 and a patchy expression of K14 in the basal epithelial cells, highlighting the undifferentiated state of the epithelium at this stage (Figures 3A–C). By 9 weeks, more K10 expressing cells were evident lying in a suprabasal layer on top of the K14 basal layer, with K5 still expressed in most cells (Figures 3D–F). By 11 weeks, expression of K10 had spread up into the VL extending from the labio-tectal furrow (Figure 3G). Expression was concentrated on the buccal side and was largely absent from the lingual side (Figures 3G,H,J), agreeing with the results of Pelissier et al. (1992). The cells of the VL near to the basal lamina and the DL expressed K14 at this stage (Figure 3G). The cells in the center of the VL did not express K14, with scattered expression of K10, indicating that the center was more differentiated than the edges (Figure 3G). On the buccal side near the oral epithelium, K14 was mainly restricted to the basal layer, with K5 expressed more widely and K10 expressed in a suprabasal layer, with some overlap of expression in the intermediate area between K14 and K10 (Figures 3H,I). On the lingual side, the expression of K14 and K5 were similar but with minimal K10, highlighting that the two sides of the lamina appear to differentiate asynchronously (Figures 3J,K).

**Figure 4:**
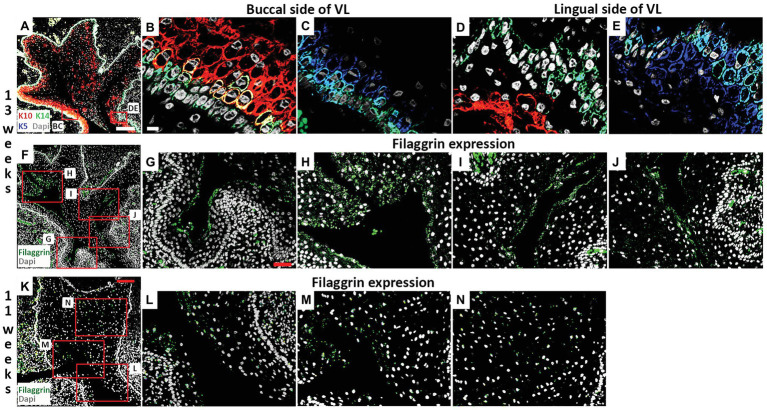
Filaggrin expression is associated with fissure formation in the VL. **(A–J)** Frontal sections through the canine VL at 13 weeks. DAPI shows nuclei in white. **(A)** Branching structure of VL. Boxes in **(A)** highlights regions in **(B–E)**. **(A–E)** K10 (red), K5 (blue), K14 (green). **(B,C)** On the buccal side, K14 is largely limited to the basal layer, with a wider expression of K5, and K10 at high levels in the overlying layer. **(D,E)** On the lingual side, K10 levels remain low, with a less organized layer of K14. **(F–J)** Filaggrin (green) lines the forming fissures at 13 weeks. **(F)** Branching structure of VL. Boxes in **(F)** highlights different parts of the VL in **(G–J)**. **(K–N)** Frontal sections through the canine VL at 11 weeks. DAPI shows nuclei in white. **(K)** VL with evidence of a main fissure running through the structure. Filaggrin (Green). Boxes in **(K)** highlights regions in **(L–N)**. Scale bar in **(A)** = 200 μm, same scale in **(F)**. Scale bar in **(B)** = 10 μm, same scale in **(C–E)**. Scale bar in **(G)** = 50 μm, same scale in **(H–J,L–N)**. Scale bar in **(K)** = 100 μm.

The strong expression of K10 on the buccal side of the VL was maintained at 13 weeks but more K10 positive cells were now found throughout the rest of the lamina (Figures 4A,B,D). The expression of K5 reduced, particularly on the buccal side, where expression was mainly restricted to the cells of the basal lamina, overlapping with K14 (Figures 4C,E). Some cells again co-expressed K14 and K10, indicating a change in differentiation state (Figure 4B). At 13 weeks, a number of fissures had developed in the VL (as seen in Figure 2H). To understand how these fissures formed, we looked at expression of filaggrin. Filaggrin has previously been reported as turning on at 22–24 weeks in the interfollicular epidermis and granular layer of the skin, with expression at 14 weeks in developing hair follicles (Dale et al., 1985; Lee et al., 1999). At 13 weeks, filaggrin was strongly expressed in the cells lining the developing fissures (Figures 4F–J). As filaggrin expression in keratinocytes results in loss of cell-cell adhesion (Presland et al., 2001), filaggrin upregulation in the VL might trigger fissure formation. To investigate this further, we analyzed filaggrin expression at 11 weeks (Figures 4K–N). As at 13 weeks, in areas where fissures had already started to form, filaggrin was expressed (Figures 4K–M). Interestingly, however, diffuse expression was also evident in the middle of the VL in regions where fissure formation was yet to initiate (Figure 4N), highlighting that filaggrin might be playing a role in fissure initiation. Filaggrin expression in keratinocytes results in decreased proliferation (Presland et al., 2001) and increased susceptibility to apoptosis (Kuechle et al., 2000). Given the links with proliferation and apoptosis, we next investigated how these processes were altered during fissure formation during VL development.

### Cells at the Center of the VL Do Not Proliferate and Undergo Cell Death

**Figure 5:**
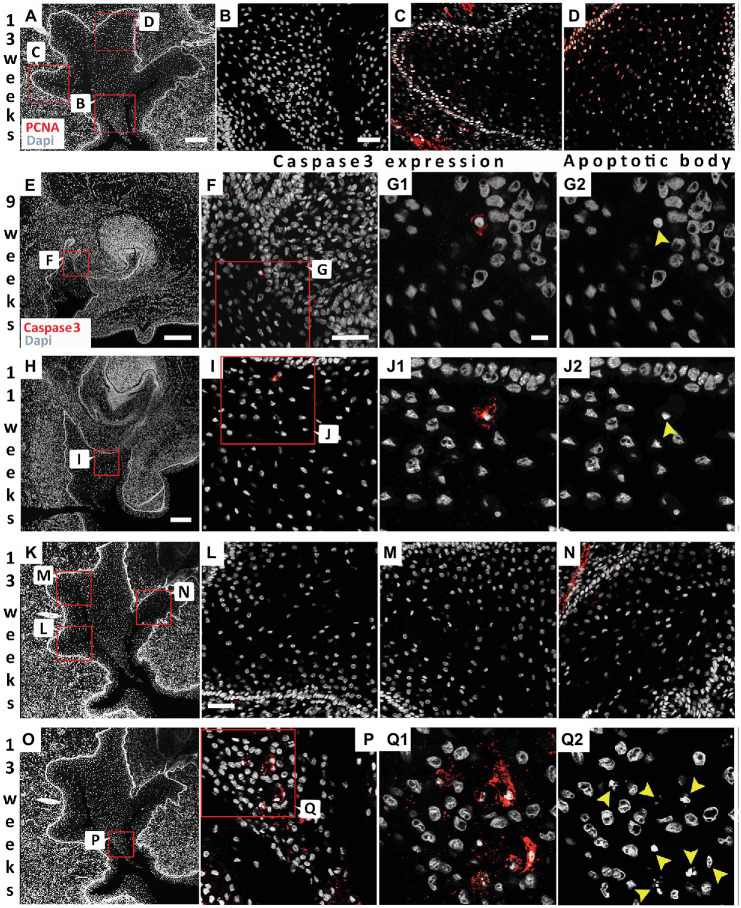
Differential proliferation and apoptosis to remove the central cells to create the vestibule. **(A)** 13 weeks VL. DAPI shows nuclei in white. Boxes in **(A)** highlights regions in **(B–D)**. **(B–D)** PCNA (red). **(B)** Absence of proliferating cells at the center of the VL. **(C,D)** Proliferating cells in the more basal cells at the edges of the VL. **(E–Q2)** Analysis of program cell death. **(F,G1,I,J1,L–N,P,Q1)** activated Caspase-3 immuno (red). **(G2,J2,Q2)** same sections as in **(G1,J1,Q1)** showing the presence of apototic bodies (yellow arrowheads). **(E–G2)** 9 weeks. **(E)** 9 weeks VL. DAPI shows nuclei in white. Box in **(E)** highlights regions in **(F–G2)**. **(H–J2)** 11 weeks. **(H)** 11 weeks VL. DAPI shows nuclei in white. Box in **(H)** highlights regions in **(I–J2)**. **(K–Q2)** 13 weeks. **(K)** 13 weeks VL. DAPI shows nuclei in white. Boxes in **(K)** highlights regions in **(L–N)**. Box in **(O)** highlights regions in **(P–Q2)**. Scale bar in **(A)** = 200 μm, Scale Bar in **(B)** = 50 μm, same scale in **(C,D)**. Scale bar in **(E,H)** = 200 μm, same scale in **(K,O)** as for **(H)**. Scale bar in **(F)** = 50 μm, same scale in **(I)**. Scale bar in **(G1)** = 10 μm, same scale in **(G2,J1,J2,Q1,Q2)**. **(G1)** = 10 μm, same scale in **(G2,J1,J2,Q1,Q2)**. Scale bar in **(L)** = 50 μm, same scale in **(M,N)**.

To investigate proliferation levels, we utilized PCNA as a marker for the S phase of cell proliferation (Dietrich, 1993). At 13 weeks, proliferating cells were generally found associated with the basal layer next to the basal lamina (Figures 5A,C,D). Fewer positive cells were observed moving away from the basal lamina (Figures 5C,D), with no positive cells in the center (Figures 5A,B). Cell death has previously been investigated during the development of the vole VL (Witter et al., 2005). A few scattered apoptotic bodies were evident in the forming VL at E13.5 and E14.5 in the vole, in contrast to high levels in the forming EKs in the DL, suggesting that cell death only played a minor role at these stages (Witter et al., 2005). A lack of degenerating cells in the VL was also described during formation of the furrow in human fetal samples (Coslet and Cohen, 1969), although others have reported localized cellular atrophy as the cause of the split (Bolk, 1921; West, 1924). We utilized Caspase-3 as a marker for apoptosis and correlated to the presence of apoptotic bodies, as identified by condensed nuclei. As in the vole, very few Caspase positive cells were observed at early stages of VL formation (9 and 11 weeks; Figures 5E–J). The few positive cells, corresponded to apoptotic bodies, confirming that the cells were undergoing programmed cell death (Figures 5G,J). At 13 weeks, there were no positive cells associated with the forming fissures (Figures 5K–N); however, a large number of positive cells were found in the V-shaped epithelial tissue at the center of the VL (Figures 5O–Q2). Cell death, therefore, appears to play a role in removal of the tissue lying in between the forming fissures but did not play a role in the formation of the fissures themselves.

## Discussion

### A Single Origin of the Anterior VL and DL During Human Development

The DL and VL have a very close relationship during development. In the maxillary canine region, the DL is physically attached to the VL at all stages investigated. In the lower anterior region, our culture experiments confirm that a single epithelial thickening gives rise to both the VL and the DL. Due to the rarity of the material, culture was only attempted for one embryo at CS19 but the results agree with findings indicated from 3D reconstruction of human sections and from lineage tracing in the mouse (Hovorakova et al., 2007, 2016). Although we confirm this dual origin for the lower anterior oral region, the relationship between the DL and VL is dynamic throughout the jaw (Figures 1A, 2A). This heterogeneity explains the differing results from papers that have investigated different regions of the jaw (Bolk, 1921; Schour, 1929; Tonge, 1969). We, therefore, suggest that a single thickening, which subsequently divides into the DL and VL, forms in the anterior region, while distinct DL and VL thickenings arise more posteriorly. Whether this is a feature across mammals, will be an interesting area for future investigation.

The DL is closely associated with a number of developing structures across vertebrates. In the mouse, a single thickened epithelium has been proposed to be the primordium for the DL, VL, and the rugae of the palate (Peterková, 1985). Similarly, in reptiles the DL and the neighboring dental gland have been suggested to form from a single placode (Vonk et al., 2008; Tucker, 2010). A close relationship between the DL and the neighboring taste bud primordium has also been suggested in the shark and rays, with cells from the taste buds contributing to the cells of the lamina during embryonic development (Martin et al., 2016). The DL should therefore not be thought of in isolation but together with its neighboring epithelial organs. What signals govern the decision to form a VL or DL is an interesting future question. Mistakes in such a process might lead to the VL taking on a DL fate, as in the case when tooth pathologies form in the region of the vestibule (Hovorakova et al., 2020).

### The Lingual and Labial/Buccal Sides of the VL Have Different Differentiation Patterns

The VL forms as a block of epithelium which then divides to create the vestibule. Here, we show that the canine VL is not divided by formation of a single fissure but by the development of numerous fissures that split the VL into several pieces. The epithelial cells on the labial/buccal side differentiate earlier, with the expression of the suprabasal marker K10, while this marker was only weakly expressed on the lingual side by 13 weeks when the fissures are apparent. Differences in keratin patterns between the mucosa on either side of the vestibule have also been observed in adult tissue, suggesting that these developmental differences are maintained throughout life (Verlach et al., 2017). It has been proposed that the lingual and labial/buccal sides are divided along the line of the K10 positive and negative expressing cells (Pelissier et al., 1992). There appears to be a slight oversimplification given that many fissures form and do not follow the lines of K10 expression. However, the fissures were lined with the terminal marker filaggrin, with filaggrin expression evident before fissure formation at 11 weeks. Filaggrin has been shown to have a role in cell adhesion (Presland et al., 2001). In epithelial cells, over expressing filaggrin, two desmosome proteins, desmoplakin, and plakoglobin, were lost at the cell interfaces and the cells detached from their neighbors (Presland et al., 2001). The upregulation of filaggrin in the VL, prior to any evidence of a fissure, may therefore lead to the cells at the center losing their adhesion, with the consequence that gaps would appear between the cells, creating the fissures. This is a particularly interesting hypothesis, given that filaggrin expression is observed extremely early in the VL, several weeks before its described upregulation in forming hair follicles and its expression in fetal skin (Dale et al., 1985; Lee et al., 1999). Division of the VL based on changes in cell adhesion agrees with the hypothesis suggested by Coslet and Cohen (1969) based on cell morphology in histological sections. The upregulation of filaggrin and differentiation of the VL cells agreed with the restriction of proliferation to the more basal parts of the structure. Failure in formation of these fissures along the VL would be predicted to result in the formation of frenulum, with tissue permanently linking the dental arch and lips/cheeks, as observed in EVC syndrome (Sasalawad et al., 2013). EVC syndrome is associated with defects in primary cilia and the Shh signaling pathway (Caparrós-Martín et al., 2013; Nakatomi et al., 2013), suggesting that this pathway may have an important role in development of the VL. In mice, the DL and VL form from a Shh positive placode but then Shh turns off in the VL (Hovorakova et al., 2016). Shh, therefore does not appear to be associated with later development of the VL.

### Apoptosis Plays a Role in Removing Tissue From the VL but Not in Fissure Formation

During formation of the fissures, we did not see any evidence of programmed cell death, as evidenced by expression of activated Caspase-3 or of apoptotic bodies. Fissure formation is therefore unlikely to be triggered by death of cells at the center of each fissure, but rather by changes in cell-cell interactions. Several fissures were formed in the VL at the canine, creating a V-shaped wedge of cells at the middle of the VL. It is here that high levels of apoptosis were observed. Removal of the cells in the middle of the VL, created by formation of the fissures, therefore does appear to be dependent on cell death. Cell death in this region would help to broaden the developing vestibule, creating a more pronounced division between the dental arch and surrounding cheeks and lips.

Overall, we show that the DL and VL develop in close association, with the VL as a transient structure, having a perhaps surprisingly complex development. New understanding of development can shed light on dental pathologies associated with the vestibule, and to congenital defects in this region.

## Data Availability Statement

The raw data supporting the conclusions of this article will be made available by the authors, without undue reservation.

## Ethics Statement

The studies involving human embryonic and fetal tissue were reviewed and approved by the Human Developmental Biology Resource under the approval of the National Research Ethics Service.

## Author Contributions

MH and AT conceived the idea. TQ performed the histology and immunohistochemistry. TT performed the explant culture experiments. AT and TQ wrote the manuscript. All authors contributed to the article and approved the submitted version.

## Conflict of Interest

The authors declare that the research was conducted in the absence of any commercial or financial relationships that could be construed as a potential conflict of interest.
